# Hyperspectral Imaging for Foreign Matter Detection in Foods: Advances, Challenges, and Future Directions

**DOI:** 10.3390/foods14173026

**Published:** 2025-08-28

**Authors:** Wenlong Li, Yuqing Wu, Liuzi Du, Xianwen Shang, Jiyong Shi

**Affiliations:** Agricultural Product Processing and Storage Lab, School of Food and Biological Engineering, Jiangsu University, Zhenjiang 212013, China; 18896656873@163.com (W.L.); 2112318154@stmail.ujs.edu.cn (Y.W.); duliuzi@163.com (L.D.); sxw18906320352@163.com (X.S.)

**Keywords:** hyperspectral imaging, deep learning, adulteration detection, food safety

## Abstract

The presence of foreign matter in food poses food safety issues for consumers and directly threatens the food supply chain. In order to ensure food quality and hygiene, promote economic efficiency, and protect consumers’ health rights, the rapid, non-destructive detection of foreign matter in food is an urgent task that requires development. Hyperspectral imaging technology can obtain high-resolution spectral information of foreign matter in multiple wavelengths, and it is widely used in food safety testing. However, the cost and size of the system remain obstacles to further development. Additionally, there are currently no effective solutions for acquiring foreign matter samples or for storing and sharing hyperspectral data during production. This review introduces hyperspectral imaging systems, covering both the software and hardware, as well as a series of algorithms for processing spectral images. The focus is on cases of hyperspectral imaging used for foreign matter detection tasks, with an examination of future developments and challenges.

## 1. Introduction

Today’s human health is associated with food safety, and it is well known that dietary-related diseases are becoming more common. Detecting foreign matters in food is critical to ensuring food safety and consumer health and improving food economics. Foreign matters are those physical, biological, and/or chemical hazards that unintentionally enter the food production stream and must be minimized. Biological hazards include bacteria, viruses, or other natural factors that may increase the risk of food safety. Contaminants such as plastics, metals, glass, and organics can pose serious health risks once they enter the food supply chain. As the global food trade continues to expand, the need for efficient and reliable detection systems has become more urgent.

A range of technologies have been used to address the problem of foreign matters detection in different types of food. These include metal detectors, X-ray detection, color imaging (optical detectors), microwave imaging, terahertz imaging, ultrasound imaging, and thermal imaging. Detection techniques based on chemical analytical methods such as gas chromatography (GC) [1], high-performance thin-layer liquid chromatography (HPTLC), high-performance liquid chromatography (HPLC) [2], mass spectrometry (MS), and polymerase chain reaction (PCR) [3] can detect foreign matters in the chemical structure of food Although these techniques are considered standard and error-free, they are highly destructive, labor-intensive, costly, and require expertise in analytical procedures, single-point detection, and long detection cycles, which has limited their use. Considering the drawbacks of destructive methods, they are not suitable for applications in rapid analyses, such as on-line mass assessment and determination in scale-up and industrial applications. At the same time, some destructive methods can only be applied on a laboratory scale as a requirement for specific instrumentation during mass analysis. In summary, the traditional methods for detecting foreign matters in food are largely subjective, labor-intensive, and destructive, whereas hyperspectral imaging is a detection technology available to non-professional inspectors for rapid and non-destructive testing of food, which not only acquires data under hundreds of consecutive wavelength bands of the sample, but also provides reflectance or radiant brightness values of the sample in each wavelength band, and has the advantage of simultaneously acquiring the spectral data and image information of the sample. Therefore, research related to the use of hyperspectral techniques to address the detection of foreign matters in food has received extensive attention in recent years.

The hyperspectral imaging system is composed of two parts, hardware and software, where the hardware includes light source, spectrometer, Charge-coupled Device(CCD) camera, etc., which work together to realize the acquisition of hyperspectral data, and the acquired spectral data is processed by the software (image processing and data analysis tools), which can be used to effectively establish the detection model of foreign matters in food. Spectral hardware equipment directly determines the quality and analyzability of the collected spectral data, while image processing and data analysis such as preprocessing, sample sets division, outlier removal, feature screening and model building steps significantly determine the robustness of the foreign matter detection model. With the advent of the big data era, the methods for building hyperspectral foreign matter recognition models are no longer limited to traditional machine learning methods, but deep learning networks such as the convolutional neural network (CNN) [4], long short-term memory (LSTM) [5], Three-Dimensional Convolutional Neural Network (3D-CNN) [6], etc., are used in the construction of models. Therefore, choosing the appropriate hardware and proficiently using the analysis software are crucial to deeply mastering the hyperspectral imaging technology. The aim of this review is to summarize comprehensive information about hyperspectral imaging in food foreign matter detection for non-spectral professionals. It provides a brief introduction to hyperspectral imaging systems, discusses model construction methods and specific applications of hyperspectral imaging for foreign matter detection, as well as describing the advantages of the technology and prospects for future research. The advantages and disadvantages of different food quality testing technologies are compared in Table 1.

## 2. Principles of Hyperspectral Imaging System

The hyperspectral imaging system integrates a spectroscopy device and an imaging system to decompose complex optical signals into successive wavelengths and convert the optical signals into electrical signals by sensing devices in order to obtain three-dimensional hypercube data at each pixel point of the sample, which contains the two-dimensional spatial information of the sample in multiple wavelength bands. And the two-dimensional spatial information is stitched into a continuous band image in the one-dimensional band dimension. The principal part of the system usually consists of an emitting light source, a wavelength dispersion device, an imaging sensor (usually a CCD camera or a Complementary Metal-Oxide-Semiconductor (CMOS) sensor) [13], and a computer equipped with image acquisition software, supplemented by a corresponding platform or carrier according to the different acquisition scenarios.

### 2.1. Light Sources and Dispersion Devices

In the scenario of detecting foreign matters in food, the differences between food and foreign matters are usually related to the physical properties (e.g., texture, color characteristics) or chemical properties (including the molecular structure of the food components) of the food samples, and the hyperspectral imaging technology offers a highly promising option for food quality inspection. The region of interest is selected according to the respective properties of the food and foreign matters being examined, which can be accurately distinguished by using different bands of the electromagnetic spectrum.

The tungsten halogen lamp is often used as a light source in hyperspectral systems. Compared with light sources such as light-emitting diodes (LEDs), lasers, and xenon lamps, tungsten halogen lamps can provide a wider spectral coverage (300–2500 nm) and constant distribution of spectral wavelengths, with the advantages of high stability, long service life, and low cost. Meanwhile visible (380–780 nm) and near-infrared wavelengths (780–2500 nm) are also the frequently used electromagnetic bands, where the reflectance information of the spectra in the visible wavelengths contains the samples’ information in the RGB color space, and the near-infrared wavelengths can accurately capture the different features of the foods related to the presences of some hydrogen containing functional groups (e.g., -OH, -NH, -CH, and -SH). In addition the ultraviolet (UV) spectrum (10–380 nm) [14] is also efficient in providing information on the chemical structure and molecular features of foods, especially in terms of conjugated and non-conjugated chemical bonds, ring structure, π-electrons, etc. [15]

Generally the light source in the hyperspectral imaging system can provide a wide band of continuous spectrum from visible to near infrared, but its output is continuous, so wavelength dispersing devices (e.g., grating, prism) are usually used to dispersed the white light originating from tungsten halogen lamps by physical principles (diffraction) to produce beams of different wavelengths. These dispersed beams are then directed at the target object or sample and the signals reflected or transmitted are captured and analyzed by sensors (e.g., CCD or CMOS detectors). The selection of a wavelength dispersing device according to the usage scenario and cost is therefore an essential step.

### 2.2. Imaging Sensors

Based on the way of acquiring data by the spectral camera, hyperspectral scanning modes can be categorized into point scanning, line scanning, surface scanning, and one-shot procedures. The point scanning, by scanning point by point, can accurately acquire the spectral data of each pixel point of the sample, providing a high spectral resolution, but requiring a camera with a high freedom of movement and higher time cost. The surface scanning camera captures the spectral data of the whole scene at one time by a two-dimensional sensor array [16], which is able to obtain the full-scene image quickly; however, for the method of capturing the spectral data of multiple pixel points at the same time, a part of spectral resolution may be lost, and there are problems such as spatial aberrations or distortions. In contrast, line-scan cameras are frequently used in food foreign matter detection [17], This scanning method only needs to control the mechanical movement of the sample in a fixed direction, and the hyperspectral data of the sample is collected and combined line by line [18], which greatly reduces the cost of filming while acquiring hyperspectral information with good resolution.

Depending on the hyperspectral imaging mode, it can be categorized into reflectance and transmission modes [19]. In reflectance mode, the light source and spectral camera are usually located on the same side of the sample and are used to evaluate sample surface features such as texture information, surface defects, and foreign matter identification. In transmission mode, the light source and CCD camera are located on opposite sides of the sample and are used to detect problems such as internal tissue, foreign matter mixing, and tissue defects [20].

### 2.3. Software

Since hyperspectral data contain much more important food information than background information, it is significant to select an appropriate region of interest (ROI) to improve the signal-to-noise ratio [21], reduce the data dimensionality, eliminate the interference of irrelevant components, and save the computational resources. The determination of ROI is usually determined by the research purpose, such as defect and foreign matter recognition, texture feature extraction, etc.

ROI extraction methods are usually divided into two modes: manual and automatic [22], in which manual ROI extraction relies on manual experience and research purposes, and interactive tools (e.g., the Environment for Visualization of Images (ENVI) software) are used to accurately annotate target regions in hyperspectral images and precisely eliminate some irrelevant background data. However, manual extraction requires more time cost [23]. Automatic extraction methods identify target regions from hyperspectral data cubes by algorithms, and their core principles are based on the joint analysis of spectral features, spatial patterns and statistical laws. However, for the problem of foreign matter recognition in food, the information distinction between the sample and the background is difficult to judge based on the pattern of automatic ROI extraction. After selecting the ROI, the average reflection value of each pixel point in the region at each wavelength can be regarded as the new feature value at the corresponding wavelength and rearranged into a combination as the average spectrum of the selected ROI region for analysis [24].

## 3. Analytical Methods in Hyperspectral Techniques

### 3.1. Preprocessing Methods

Processing of hyperspectral data usually involves a series of steps, including image acquisition and calibration, extraction of spectral data, data preprocessing, screening of characteristic bands, spectral data modeling, and image visualization. The acquired hyperspectral image can be regarded as a three-dimensional data cube storing a two-dimensional spatial dimension and a one-dimensional spectral dimension, which contains not only the spectral data of the samples, but also redundant noise and background information, which is usually due to the acquisition environment and the human factors, causing a great deal of trouble to the processing of spectral data [25].

In analyzing and processing spectral data, different preprocessing methods are usually used to remove noise, enhance features, and correct bias from the raw spectral data for subsequent data analysis and model building. The commonly used preprocessing methods include standard normal variate transformation (SNVT) [26], multiple scattering correction (MSC) [27], mean centralization (MC), first derivative (1st Der) [28], second derivative (2nd Der) [29], and convolutional smoothing (Savitzky–Golay, SG) [30], in which SNVT is the conversion of any continuous random variable to a standard normal distribution (normal distribution with mean 0 and standard deviation 1), helping to reduce variations between different experimental conditions, instruments, or samples and leading to better highlighting of chemical properties in the spectra; the method is stronger in eliminating the effect of sample particle size and baseline drift. The idea of MSC is to take the average of all spectral data as the ideal spectrum, and analyze the baseline shift and offset of each sample by one-way linear regression, to effectively eliminate the spectral differences due to different scattering levels, thus enhancing the correlation between the spectra and the data [31]. The MC process is based on the idea of subtracting the mean from each data matrix and adjusting each data point in the dataset to have a mean of zero relative to the dataset. This helps to remove the overall bias in the data in order to better understand the relative variation and distribution of the data. First derivatives eliminate the translation of the spectral baseline, i.e., removing the fixed deviation that exists between the absorbance and the true value, and second derivative processing removes the rotation of the baseline, whereas derivative computation tends to add unnecessary noisy information, so smoothing is often used in conjunction with derivative preprocessing.

### 3.2. Data Dimensioning and Compression

Variable screening and feature extraction are both methods of downgrading and compressing raw data, which can eliminate information that is not relevant to the object to be measured and simplify the modeling process [32]. The difference between the methods is that the variable screening is a specific method to select the most effective part of the variables from the acquired data of the sensor to simplify the process of feature extraction, improve the computational efficiency, and further establish a mathematical model with strong predictive ability and good stability. Feature extraction restructures the original data by mapping or transforming and extracts new features for further processing and analysis. When analyzing spectral data, feature extraction is usually conducted after using variable screening methods, and the extracted features are finally used to build mathematical models.

Currently the commonly used variable screening methods are the interval screening method based on the composition of the sample set and methods based on filtering, wrapping, and embedding ideas in machine learning applications, such as Uninformative Variables Elimination (UVE) [33], the genetic algorithm (GA), competitive adaptive reweighted sampling (CARS) [34], and the sequential projection algorithm (SPA) [35].

The CARS algorithm is a feature variable selection method that combines Monte Carlo sampling with partial least squares (PLS) [36] model regression coefficients to efficiently extract representative bands from high-dimensional data and avoid redundant data, but it requires a large number of calculations and iterations, and sometimes falls into local optimal solutions. SPA belongs to the unsupervised learning process, which is a forward iterative search method that starts from a wavelength and adds a new variable during each iteration until the number of variables reaches a set threshold. The algorithm is relatively simple, and it is easy to ignore some complex, high-order features when there is a non-linear relationship between the features. The core of UVE is adding a matrix of random variables (noise) with the same number of variables as the independent variable matrix within the original spectral data, and a new independent variable is composed based on the spectral variables and the noise. The matrix makes a variable judgement on the statistical distribution of the regression coefficients of the target matrix. The algorithm relies on a certain threshold setting, and the choice of the threshold may affect the final feature selection results. The idea of GA originates from the evolutionary process of biological species in nature, interpreting the objective function to be optimized as the adaptability of the biological population to the environment. As a global optimization algorithm, GA can effectively avoid the global optimal solution and adapt to non-linear and unstable data.

The screening of the above algorithms may ignore the key information embedded in the complex spectral–spatial relationship; in order to solve this limitation, there are more feature engineering methods for extracting discriminative information from hyperspectral datasets, especially in the identification of foreign matters in food, such as Grey-Scale Covariance Matrix (GLCM) [37], principal component analysis (PCA) [38], convolutional neural network (CNN) [39], and Independent Component Analysis (ICA) [40].

GLCM extracts second-order statistical texture features (e.g., contrast, correlation, energy) by calculating the joint probability distribution of pixel-pair grey values for specific spatial relationships (e.g., distance, orientation) in an image. It can effectively characterize the texture features of the sample. PCA preserves the principal components in the direction of maximum variance by orthogonal transforming the high-dimension spectral data projected to a low-dimension space. It can effectively eliminate the redundant noise information between bands and compress hundreds of bands to 3–5 principal components, which greatly reduces the computational cost. CNNs have the ability to automatically learn spectrum–space association for feature extraction by applying multi-layer convolution-pooling operation to the data. However, the performance is highly dependent on the hyperspectral data scale, computational resources, and field adaptability of the network design. ICA is based on the principle of assuming that the observed signals are linear mixtures of independent sources, and by optimizing statistical independence to separate the source signals, it is particularly suitable for scenarios where there is a lot of noise or redundant information. It can effectively improve the accuracy of data analysis by separating independent components and removing redundant information. However, ICA is not applicable to all types of hyperspectral data due to the high computational complexity and the requirement of data independence.

**Figure 1:**
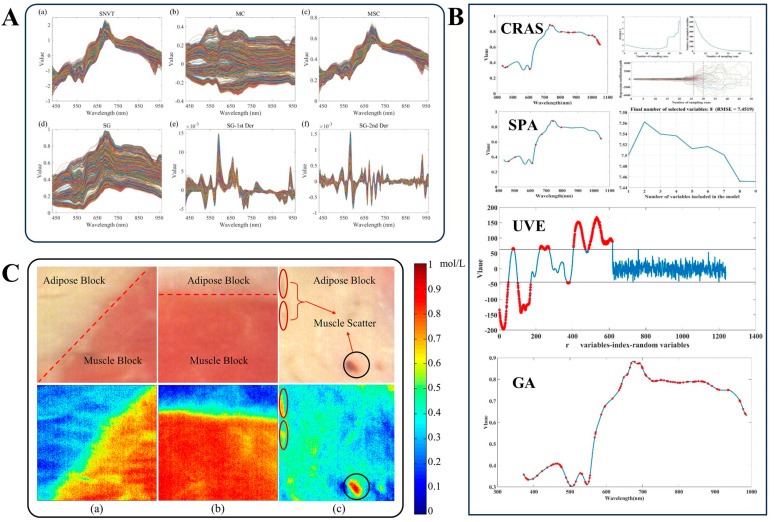
**Application of preprocessing and data compression methods in hyperspectral data**. (**A**) (**a**) SNVT, (**b**) MC, (**c**) MSC, (**d**) SG, (**e**) 1st Der, and (**f**) 2nd Der for spectral data processing. (**B**) CARS, SPA, UVE, and GA all reduce dimensionality and compress spectral data. Reproduced from Ref. [41]. (**C**) Visualization of the sugar content of beef, based on processed data. (**a**,**b**) Adipose tissue and muscle tissue; (**c**) Adipose tissue interspersed with other tissues. Reproduced with permission from Ref. [42]. Copyright 2023 Elsevier.

Hyperspectral data compression typically involves the filtering or construction of features to reduce the data dimensionality while retaining key information. This process not only improves the computational efficiency of existing models, but also enhances their robustness. Therefore, the adoption of feature selection or feature construction methods is crucial for hyperspectral data compression, which can help to enhance data processing capabilities and optimize the performance of subsequent analysis tasks. Figure 1A,B demonstrate how various preprocessing and data compression methods can be applied to hyperspectral data to visualize food indicators. As shown in Figure 1C, the application of processed hyperspectral data can be used to determine the sucrose content in beef muscle and adipose tissue.

### 3.3. Spectral Modeling Methods

Hyperspectral imaging technology achieves the ‘one image, one spectrum’ description of food composition through continuous band spectral reflectance information. However, the hundreds of dimensions of spectral data and the complex spectral–spatial coupling characteristics pose the challenge of multi-dimensional interpretation for modeling methods [43]. The core of spectral modeling is to establish the mathematical mapping relationship between spectral features and target attributes (e.g., foreign matter, chemical content).

#### 3.3.1. Machine Learning Modeling Methods

The qualitative classification model is one of the traditional machine learning models that automatically detects and identifies any specific pattern or structure in the data by labeling discrete sample data points into different categories or category labels, and later assigning each data point to the corresponding category based on the features and attributes of the input objects. Pattern recognition can be categorized into three distinct classifications: supervised learning, unsupervised learning, and semi-supervised learning. In the context of supervised learning, the model is taught through the labeled training data and is used to predict or classify new data. Unsupervised learning is employed in the context of unlabeled data, while semi-supervised learning models represent a combination of the two and have the capacity to enhance model performance in specific situations. The following qualitative classification models are most commonly used: k-nearest neighbor (KNN) [44], back-propagation artificial neural network (BP-ANN) [45], support vector machine (SVM) [46], and linear discriminant analysis (LDA) [47].

KNN employs a scale of distances (Euclidian, Manhattan, and Minkowski distances, etc.) to measure the proximity between data points. The term ‘k’ is used to denote the number of samples in proximity that are to be taken into account when performing the classification process. This value is typically determined by cross-validation, where smaller values of k result in the model becoming more sensitive to noise and redundant features, which can lead to a small reduction in model accuracy, and larger values of k result in the model becoming too smooth [48].

BP-ANN typically comprises an input layer, one or more hidden layers, and an output layer, with the network trained using a back-propagation algorithm [49]. In its forward-propagation process, each neuron computes a weighted sum of the inputs, which is subsequently output by an activation function and produces a prediction [50]. The model determines whether further iterations are required by calculating the root-mean-square error between the prediction and the actual target, or the loss function, such as cross-entropy. If the error falls outside the acceptable range, the error information is propagated backwards, and the error is reduced according to the gradient descent algorithm and the adjustment of the weight parameters, and so on, until the end of the iteration.

LDA is a probabilistic generative model based on hidden variables that provides a probability distribution for the topic of each document in the entire collection [51]. The fundamental principle underlying LDA is data dimensionality reduction, which involves the classification of documents by identifying the optimal boundary between classes through the maximization of inter-class variance and the minimization of intra-class variance, facilitated by a linear discriminant function.

SVMs utilize kernel tricks (linear kernel, polynomial kernel, radial basis function kernel, etc.) to map data into a high-dimensional space, and find the parameters of the hyperplane by constructing a Lagrange function and maximizing its dual form to maximize the spacing between different classes of data points. Based on its algorithmic principles, the SVM is more sensitive to feature scales and is more robust to noisy data for better data generalization [52].

#### 3.3.2. Deep Learning Modeling Methods

With the development of artificial intelligence and big models, deep learning models show significant advantages in hyperspectral foreign matter classification tasks by their powerful feature extraction capability and adaptive learning properties for high-dimensional data [53]. The learning paradigm of deep neural networks also includes supervised, unsupervised, and semi-supervised learning: in supervised learning, the model optimizes the network parameters by using a large amount of labeled data to complete the classification or regression task; unsupervised learning focuses on mining the potential structure of unlabeled data (e.g., clustering, dimensionality reduction); semi-supervised learning combines a small amount of labeled data with a large amount of unlabeled data to improve the model’s generalization ability. Currently, the most popular deep neural network models include: CNN [54], LSTM [55] and Transformer [56] model.

The core principle of the 3D-CNN [57] is the simultaneous extraction of spatial features (local texture, shape) and spectral features (band continuity) of hyperspectral data by three-dimensional convolutional kernels (spatial dimension X × Y and spectral dimension λ). Unlike the traditional two-dimensional convolutional neural network (2D-CNN) [58], which focuses only on spatial information, the convolutional kernel of 3D-CNN covers both the spatial neighborhood of adjacent pixels and the spectral sequence of continuous bands during the sliding process. This design can effectively capture the spatial-spectral coupling characteristics in hyperspectral data, such as the spectral difference between the strong reflection of metallic foreign matters in a particular band and the surrounding background, or the local spectral absorption peak shift due to protein oxidation.

The CNN-LSTM neural network [59] is a hybrid architecture that combines the CNN and the LSTM with the objective of capturing both spatial features and temporal dependencies of hyperspectral data [60]. The local texture and spatial distribution features of the hyperspectral cube are first extracted by the CNN [61] module, and then the dynamic temporal variations of the spectral dimensions (e.g., the continuity of absorption peaks between bands or the cumulative effect of oxidative damage) are modeled by the LSTM [62] module. This joint spatial–temporal modeling mechanism enables the model to extract multi-level and multi-dimensional discriminative features from complex hyperspectral data.

**Figure 2:**
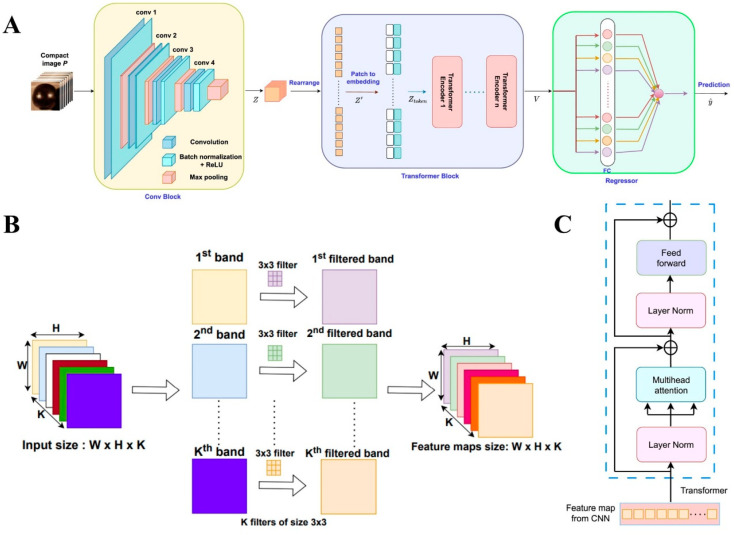
**Application of preprocessing and data compression methods in hyperspectral data.** (**A**) CNN and Transformer Composite Network. Reproduced with permission from Ref. [65]. Copyright 2025 Elsevier. (**B**) Diagram showing spatial convolution operations on hyperspectral data. Reproduced with permission from Ref. [66]. Copyright 2025 Elsevier. (**C**) Transformer block. Reproduced with permission from Ref. [65]. Copyright 2025 Elsevier.

Transformer is a deep learning architecture based on the self-attention mechanism, which was initially applied in the field of Natural Language Processing (NLP) [63], and in recent years has demonstrated excellent performance in hyperspectral image processing. The fundamental principle of Transformer is to dynamically capture long-range dependencies in data through global dependency modeling, thus eliminating the local sense-field limitation of traditional convolutional or cyclic structures. In hyperspectral data, Transformer [64] analyses the non-linear relationships between bands and spatial pixels in parallel through multiple sets of self-attentive heads to achieve end-to-end feature learning. Figure 2 shows the specific framework and calculation methods of CNN and Transformer.

#### 3.3.3. Model Assessment Parameters

The following metrics are commonly used in machine learning models to evaluate model performance and generalization ability: Root Mean Square Error of Cross-validation (RMSECV) [67], which is mainly applied to evaluate the feasibility of a certain modeling method and the predictive ability of modeling results. This metric is calculated during the training phase of a model by means of an interactive validation method. Root Mean Square Error of Prediction (RMSEP) [68] is a metric used to evaluate the predictive capability of a constructed model when applied to external samples. A lower RMSEP value indicates a higher predictive capacity of the model. The Coefficient of Determination (R^2^) is a statistical measure used to assess the model’s capacity to explain the variability in the data. It reflects the extent to which the predicted values align with the true values. A value closer to 1 indicates a stronger model fit, suggesting that the model is able to effectively explain the data.

## 4. Applications for Foreign Matter Detection

HSI has become a key tool in food foreign matter detection due to its unique ability to combine spectral and spatial information. By capturing the spectral characteristics of a specific region pixel by pixel in a continuous band, this technique can accurately identify foreign matters in food [69], while achieving non-destructive detection and rapid screening. However, due to the complexity of the food matrix and the diversity of foreign matters, researchers need to experimentally compare different combinations of spectral wavelength ranges, feature extraction algorithms, and classification models to optimize the detection accuracy and efficiency. Since there is no uniform standard for parameter selection in the spectral detection process, this paper summarizes the technical adaptation rules in different application scenarios by systematically combing typical cases and provides non-professional hyperspectral researchers with a relocatable methodological framework and decision-making basis to promote the standardized application of hyperspectral imaging in foreign matter detection. Table 2 summarizes the data processing methods and performance of the various hyperspectral imaging systems employed for detecting foreign matter in meat, plant-based foods, and other foods.

### 4.1. Detection of Foreign Matters in Meat Foods

Meat foods are rich in fat, protein, iron, zinc, selenium, glutathione, and many vitamins [70]. They are important sources of nutrition for people, so the method of accurately safeguarding meat foods has received a lot of attention [71]. Hyperspectral imaging, based on the short-wave infrared (SWIR) [72] spectrum, has been utilized to evaluate the extent of pork adulteration in beef and lamb food products. The system employs data preprocessing methodologies, including normalization, smoothing, SG-1st Der, and SG-2nd. It subsequently establishes a partial least squares regression (PLSR) [73] discriminative model following PCA compression of the data dimensions. This enables the accurate identification and determination of pork adulteration in beef and lamb meat.

Another hyperspectral system successfully detected single-component and multi-component mixed adulteration in marine fishmeal. This was achieved by implementing baseline correction methods, such as standard normal variable (SNV), 1st-Der [74], and 2nd-Der. Additionally, data compression techniques, including PCA, CARS, and SPA, as well as partial least squares-discriminant analysis (PLS-DA) [75] and SVM modeling methods were employed. The results indicate that the quality of marine fishmeal detection can be effectively managed, and that powder foreign matter adulteration can be accurately identified through the selection of appropriate preprocessing, data compression, and modeling methods.

A transmission-mode hyperspectral imaging system has been utilized for the identification of foreign matters of similar density, including glass, iron, plastic, and silica gel, which have been embedded within soy protein meat semi-finished products (SFSPM). Noise and interferences in the raw spectral data can be effectively removed by preprocessing such as SNVT, SG, 1st-Der, 2nd-Der, and MSC. The feature band screening methods such as PCA, CARS [76], and SPA are combined with KNN, BP-ANN, SVM, and LDA algorithms for modeling. The findings indicate that the transmission-mode based hyperspectral imaging technique performs significantly better than ultrasound imaging and computer vision methods in the separation of analogous density foreign matters in SFSPM.

Near-infrared (NIR) hyperspectral imaging [77] allows for the simultaneous detection of endogenous and exogenous adulteration in minced beef, such as fraudulent substitution of minced beef with beef liver, beef heart, and pork. The system employs SNV, MSC, SG-1st-Der, and SG-2nd-Der to remove the noise, filtering the feature bands using GA and Interval partial least-squares (iPLS) [78] after dimensionality reduction by PCA, and build models including soft independent modeling by class analogy (SIMCA) and PLS-DA and non-linear algorithms (support vector machine-discriminant analysis (SVM-DA), classification and regression tree (CART) [79], and BP-ANN). The results show that the hyperspectral imaging system can be used to identify each type of adulterant in beef samples on the basis of pure beef, thus providing a more comprehensive analysis of adulteration in the meat industry.

### 4.2. Detection of Foreign Matters in Plant Foods

As an important source of vitamins, antioxidants, and dietary fiber for humans, the consumption level and safety of fruits and vegetables are directly related to global public health. However, foreign matters can easily be mixed in during agricultural production and industrial processing, thus compromising food safety and causing some economic losses, while the high spatial resolution of hyperspectral imaging can help to detect and remove foreign matters. For example, a combination of fluorescence imaging, visible near-infrared imaging, and short-wave infrared imaging with the PLS-DA model can identify a wide range of foreign matters mixed in freshly cut vegetables. The system uses a combination of preprocessing methods (normalization, MSC, SNV, SG, and derivatives) and compression methods (sequential feature selection (SFS), SPA, and iPLS) to improve the signal-to-noise ratio of the spectral data. The SPA-PLS-DA model was found to be the most effective, demonstrating that a hyperspectral system with multivariate analysis can be used to effectively identify different types of foreign matters in freshly cut vegetables.

A system for detecting adulteration in Crocus sativus, a traditional Chinese medicinal herb that is used in both medicine and food, has been developed. Hyperspectral imaging in the visible range is combined with SNV and MSC, alongside various data compression methods (PCA, PLS), to create LDA, PLS-DA, multilayer perceptron (MLP) and SVM. Due to its resilience to variable imputation methods and low sensitivity, SVM demonstrates a more reliable effect in the Crocus sativus adulteration system, producing more reliable results.

Ganoderma lucidum spore powder has important medicinal and economic values, so it is often illegally adulterated with dyed starch. A hyperspectral data fusion system in the NIR and visible ranges accurately distinguishes impurities in Ganoderma lucidum spore powder. The system combines various preprocessing methods (Gaussian Filter Smoothing (GFS), SG, normalization, SNV, MSC, 1st-Der, and 2nd-Der) and data compression methods (CARS, SPA, GA, random frog (RF), and UVE), and data fusion methods are introduced to construct the recognition models of PLS-DA and PLSR based on the fused spectral data with excellent performance. This indicates that for the foreign matter recognition task in hyperspectral imaging, a certain degree of data fusion strategy can improve the robustness and generalization performance of the recognition models.

### 4.3. Detection of Foreign Matters in Other Foods

Hyperspectral imaging is also used in a variety of other scenarios, such as the detection of foreign matters in food, including nuts, porridge, and cereals. Nuts are an important component of nutrient-dense diets, rich in high-quality unsaturated fatty acids, plant proteins, and dietary fiber, which play a key role in the maintenance of metabolic homeostasis and in the prevention and control of chronic diseases. As the nutritional integrity of nut products depends on the health of their ingredients, identifying foreign matters in nut foods is essential. The short-wave infrared range of hyperspectral imaging can distinguish multiple foreign matters mixed in cocoa beans. The system combines 2nd-Der and PCA dimensionality reduction methods to respectively construct SVM, LDA, and KNN classification models. The SVM model with the optimal wavelengths as the input feature vectors performs better, which suggests that the proper selection of the feature bands plays a crucial role in the hyperspectral classification model.

Near-infrared hyperspectral imaging is also suitable for identifying endogenous foreign matters accidentally ingested during walnut processing, which uses Detrend (DT), SG, and SNV preprocessing combined with PCA compression methods to process high-dimensional spectral data and introduces the Butterfly Optimization Algorithm (BOA) and the Efficient Channel Attention (ECA) mechanism modules on the basis of SVM, MLP, VGG16, LSTM, AlexNet, Conv3Net, and WT-NIRSNet models. The results demonstrate that deep learning can perform the task of extracting high-dimensional data features more effectively in hyperspectral qualitative scenarios.

LDA, KNN, BP-ANN, and SVM models were used for hyperspectral imaging in the visible range to differentiate homochromatic foreign matters mixed in porridge-based food products. The SNV, SG, vector normalization, 1st-Der, 2nd-Der, and MSC were used for preprocessing. The compression methods were PCA and SPA, while the best identification model was SVM, which indicates that SVM has a significant advantage in handling fusion data with high non-linear intensity.

As the core source of dietary energy in daily life, cereals play an important role in preventing and controlling metabolism-related diseases. In recent years, adulteration of cereal products has become a more frequent issue, and near-infrared hyperspectral imaging can accurately detect peanut flour adulteration in whole wheat flour. The baseline correction (BLC), normalization, SNV, MSC, 1st-Der, and 2nd-Der methods are combined with the CARS data compression method to build the PLSR identification model in this system. Combining SNV and derivative preprocessing methods effectively enhances spectral data features, indicating that baseline drift and scattering effects are the main interferences in hyperspectral data for powdered grains.

**Table 2:** Application of HSI in the detection of foreign matter in food.

Purpose	Wavelength Range (nm)	Preprocessing Methods	Compression Methods	Modeling Methods	Performance	Ref.
Prediction of pork adulteration levels in minced beef and mutton samples	895–2504	SG-1st-Der, Scale, SG, NOR	PCA	PLSR	Calibration $R^{2}$ = 0.98 RMSE = 2.53 Prediction $R^{2}$ = 0.97 RMSE = 3.16	[80]
Detection of adulteration in marine fishmeal	874–1734	NOR, SNV, 1st-Der, and 2nd-Der	PCA, CARS, and SPA	PLS-DA and SVM	Three-class accuracy = 100.00% Four-class accuracy = 99.43%	[81]
Locating and separating foreign matter of similar density in semi-finished soy protein meat products	432–963	SNVT, SG, 1st-Der, 2nd-Der, and MSC	PCA, CARS, and SPA	KNN, BP-ANN, SVM, and LDA	Calibration $R^{2}$ = 96.67% Validation $R^{2}$ = 95.00%	[82]
Detection of low-level adulteration in ground beef	900–1700	SNV, MSC, SG-1st-Der, and SG-2nd-Der	PCA, GA, and iPLS	SIMCA, PLS-DA, SVM-DA. CART, and BP-ANN	Sensitivity = 1.00 Specificity = 1.00 KAPPA = 1.00	[83]
Detection of foreign matter in fresh-cut vegetables	397–2504	NOR, MSC, SNV, and SG	SFS, SPA, and iPLS	PLS-DA	Calibration $R^{2}$ = 98.60% Validation $R^{2}$ = 98.30%	[84]
Detection of adulteration in Crocus sativus saffron	900–1700	SNV and MSC	PCA and PLS	LDA, PLS-DA, MLP, and SVM	Cross-validation $R^{2}$ = 100.00% Prediction $R^{2}$ = 100.00%	[85]
Detection of foreign matter in cocoa beans	900–1700	2nd-Der	PCA	SVM, LDA, and KNN	Train $R^{2}$ = 89.10% Test $R^{2}$ = 83.96%	[86]
Detection of endogenous impurities in walnuts	855–1705	DT, SG, SNV	PCA	SVM, MLP, VGG16, LSTM, AlexNet, Conv3Net, and WT-NIRSNet	Recall = 97.44% Precision = 97.41% Accuracy = 97.45%	[87]
Separating foreign matter of the same color from mixed porridge	432–963	SG, NOR, 1st-Der, 2nd-Der and MSC	PCA and SPA	LDA, KNN, BP-ANN, and SVM	Calibration $R^{2}$ = 98.33% Validation $R^{2}$ =9 9.17%	[88]
Rapid detection of adulteration in Ganoderma lucidum spore powder	900–1700	GFS, SG, NOR, SNV, MSC, 1st-Der and 2nd-Der	CARS, SPA, GA, RF and UVE	PLS-DA and PLSR	Training set accuracy = 100.00% Prediction set accuracy = 97.14% Recall = 98.28% Precision = 92.86% F1 score = 95.49%	[89]
Detection of low-level peanut powder contamination in whole wheat flour	935–1720	BLC, NOR, SNV, MSC, 1st-Der, 2nd-Der SNV, and SNV- SG-2nd-Der	CARS	PLSR	Cross-validation $R^{2}$ = 99.40% Prediction $R^{2}$ = 99.30%	[90]

Abbreviations: Savitzky–Golay (SG), normalization (NOR), standard normal variable (SNV), first-order derivative (1st-Der), second-order derivative (2nd-Der), standard normal variate information (SNVT), multiplicative scatter correction (MSC), Detrend (DT), the baseline correction (BLC), principal component analysis (PCA), competitive adaptive reweighted sampling (CARS), sequential projection algorithm (SPA), genetic algorithm (GA), interval partial least squares (iPLS), sequential feature selection (SFS), partial least squares (PLS), random frog (RF), Uninformative Variables Elimination (UVE), partial least squares regression (PLSR), partial least squares-discriminant analysis (PLS-DA), support vector machine (SVM), k-nearest neighbor (KNN), back-propagation artificial neural networks (BP-ANNs), linear discriminant analysis (LDA), soft independent modeling by class analogy (SIMCA), classification and regression tree (CART), multilayer perceptron (MLP), Visual Geometry Group 16-layer network (VGG16), long short-term memory (LSTM).

## 5. Challenges and Future Perspectives

This review provides an overview of recent advances in hyperspectral imaging for detecting foreign matters in food products. It confirms that hyperspectral imaging has great potential as a fast, non-destructive tool for identifying and screening foreign matters. However, the implementation of this technology in industrial applications still faces many challenges.

The current hyperspectral imaging systems used in food production mainly rely on dispersive CCD cameras, which require large spectroscopic gratings and CCD sensors, resulting in high costs and large volumes [91]. To achieve deployment in industrial scenarios and improve the portability of hyperspectral imaging systems, miniaturization of the systems has become essential [91]. At present, this problem can be effectively solved using tunable photonic response and reconstruction algorithms.

The high dimensionality of hyperspectral data often causes issues such as redundant information and high computational costs. Traditional solutions rely on dimensionality reduction algorithms [92], but these can easily lose non-linear features during the dimensionality reduction process. However, with the development of artificial intelligence and increased computer processing power, a large number of deep learning algorithms can be applied to process hyperspectral data. For example, the end-to-end CNN model can map the original data directly onto the results of foreign matter detection, greatly reducing the dependence on feature engineering [93].

In the scenario of identifying foreign matters in food, hyperspectral data of normal food can be acquired at scale through production lines. However, the scarcity of foreign matter samples and the imbalance in data distribution still limit the application of hyperspectral imaging technology on a large scale. Firstly, the heterogeneity of foreign matters and random contamination scenarios make real foreign matter samples extremely difficult to collect, while labeling is cumbersome and costly. On the other hand, the collected data is unevenly distributed. In actual production, the number of positive samples (food samples containing foreign matters) is often much smaller than the number of negative samples (normal food samples). This imbalance in sample categories leads to the model being over-fitted to the characteristics of negative samples, which seriously reduces its generalization ability. To address the problem of scarce sample data, transfer learning can significantly reduce the need for labeled samples by exploiting the generic features of pre-trained models. The small-sample learning strategy can rapidly train the model to recognize new classes of foreign matters using a limited number of samples, making it highly adaptable to foreign matters with diverse morphologies.

The final challenge is the storage and sharing of hyperspectral data. To accurately identify spectral features related to foreign matters, compositional changes, contamination levels and other food quality factors in hyperspectral data, a large amount of annotated hyperspectral data is needed. This includes information about the food itself (e.g., type of substrate and physicochemical properties) and the foreign matters (e.g., dimensions, locations, and spectral features). Therefore, establishing hyperspectral data storage standards and supporting fast retrieval are prerequisites for effective utilization of the data. To avoid the repeated collection of similar foreign matter samples from food by researchers, and to optimize the recognition model by pooling resources, a hyperspectral data sharing mechanism is crucial. However, data sharing still faces problems caused by differences in hardware and software formats. In the future, it will be necessary to build an open, collaborative hyperspectral data platform. This will unify data annotation specifications and enable collaborative training of multi-source data. This will maximize the value of shared data and promote rapid iteration of foreign matter identification models in food.

## 6. Conclusions

The globalization of the food supply chain means that contamination by foreign matters in food can directly lead to issues of consumer safety. In this review, we introduce the optical device and imaging system of a hyperspectral imaging system for the rapid, non-destructive detection of mixed foreign matters in food. We also discuss in detail the methods of analyzing hyperspectral data, such as machine learning and deep neural networks. Additionally, we compile a substantial body of literature on the use of hyperspectral imaging for detecting and identifying foreign matters in food, with a particular focus on meat, plant-based, and nut-based products. Although this paper focuses on foreign matters in food, hyperspectral imaging has a wide range of potential applications in agriculture, environmental monitoring, and medical diagnosis [94]. Currently, the high processing cost of hyperspectral data and the large size of the required hardware are the main challenges hindering further development. To address these issues, developing micro-spectral technology and deep learning algorithms could facilitate the widespread adoption of hyperspectral imaging technology for identifying foreign matters in food, providing a more efficient and convenient solution for ensuring food safety and security.

**Literature Review Methodology**

**1. Database sources:**

In order to provide comprehensive coverage of research on hyperspectral imaging in the field of food foreign object detection, we searched the following academic databases:

• Web of Science

• Elsevier

• Nature

• Science

• China National Knowledge Infrastructure (CNKI).

These databases cover interdisciplinary fields such as food science, spectral imaging and agricultural engineering, ensuring the literature sources are comprehensive and authoritative.

**Keyword Strategy**

**2. A combined keyword search strategy was employed:**

(‘hyperspectral imaging’ OR ‘HSI’) AND (‘food safety’ OR ‘food quality’) AND (‘foreign matter’ OR ‘contaminant’ OR ‘adulteration’)

**3. Year range and document type**

Time span: January 2015–January 2025 (covering a period of rapid technological development over the past 10 years).

## Figures and Tables

**Table 1:** Quality control technologies employed in the food industry.

Technology	Advantage	Disadvantage	Ref.
Hyperspectral imaging	Non-destructive High-dimensional information	High cost Data redundancy	[7]
Near-infrared imaging	Portable devices are inexpensive. Non-destructive	Surface inspection only Available only for specific wavelengths	[8]
Raman spectroscopy	Rapid analysis Accurate identification of molecular structures	Expensive equipment Slow detection speed	[9]
Terahertz spectroscopy	Non-destructive Sensitive to polar molecules	Low resolution Bulky equipment that is difficult to deploy	[10]
X-ray	High-speed online inspection Strong packaging penetration	Unable to identify organic matter High misjudgment rate	[11]
Traditional chemical detection methods	High sensitivity Reliability and reproducibility	Destruction of samples High cost	[12]

## Data Availability

No new data were created or analyzed in this study. Data sharing is not applicable to this article.
